# Adverse Liver and Renal Outcomes After Initiating SGLT‐2i and GLP‐1RA Therapy Among Patients With Diabetes and MASLD

**DOI:** 10.1111/1753-0407.70069

**Published:** 2025-04-27

**Authors:** Arunkumar Krishnan, Carolin V. Schneider, Diptasree Mukherjee, Tinsay A. Woreta, Saleh A. Alqahtani

**Affiliations:** ^1^ Department of Medicine Wake Forest University School of Medicine Winston Salem North Carolina USA; ^2^ Department of Supportive Oncology Atrium Health Levine Cancer Charlotte North Carolina USA; ^3^ Department of Medicine III, Gastroenterology, Metabolic Diseases, and Intensive Care University Hospital RWTH Aachen Aachen Germany; ^4^ Department of Medicine Apex Institute of Medical Science Kolkata West Bengal India; ^5^ Division of Gastroenterology and Hepatology Johns Hopkins University School of Medicine Baltimore Maryland USA; ^6^ Organ Transplant Center of Excellence King Faisal Specialist Hospital and Research Center Riyadh Saudi Arabia; ^7^ Division of Gastroenterology and Hepatology Weill Cornell Medicine New York New York USA

**Keywords:** chronic kidney disease, cirrhosis, DPP4 inhibitors, GLP1, hepatocellular carcinoma, liver outcomes, MASLD, renal outcomes, SGLT2 inhibitors, T2DM

## Abstract

**Context:**

The management of metabolic dysfunction‐associated steatotic liver disease (MASLD) and type 2 diabetes mellitus (T2DM) presents a significant clinical challenge, with a focus on preventing progression to liver and renal complications.

**Objective:**

To evaluate the liver and renal outcomes among new users of sodium‐glucose cotransporter 2 inhibitors (SGLT2i) versus glucagon‐like peptide‐1 receptor agonists (GLP‐1RA), dipeptidyl peptidase‐4 inhibitors (DPP4i) and other anti‐diabetic medications in patients with MASLD and T2DM.

**Design:**

Retrospective cohort study.

**Setting:**

Electronic health records.

**Participants:**

A total number of 88 306 patients with MASLD and T2DM were included in a propensity score‐matched analysis comparing the effects of anti‐diabetic drugs.

**Intervention:**

Patients were categorized into groups based on their initiation of anti‐diabetic medications.

**Main Outcome Measures:**

The primary outcomes were the incidence of cirrhosis, hepatic decompensations, and hepatocellular carcinoma. Secondary outcomes were a progression of chronic kidney disease (CKD), severity of CKD stages, and the need for hemodialysis.

**Results:**

In the SGLT2i versus DPP4i, a reduced risk of cirrhosis was observed in the SGLT2i (HR: 0.97), along with fewer hepatic decompensations (HR: 0.84) and a lower incidence of HCC (HR: 0.50). CKD progression, particularly to stages 4–5, was significantly lower in the SGLT2i (HR: 0.53), as was hemodialysis (HR: 0.38). However, SGLT2i exhibited a slightly lower risk of CKD progression (HR: 0.77) and a reduced need for hemodialysis (HR: 0.71) compared to the GLP‐1RA, while there was no difference in hepatic outcomes between the GLP‐1RA and SGLT2i.

**Conclusions:**

SGLT2 inhibitors in patients with MASLD and T2DM demonstrated reduced risks of liver complications and a favorable impact on renal outcomes. These findings support the preferential consideration of SGLT2i in managing this patient population, particularly for mitigating the progression of liver and kidney diseases.

## Graphical abstract

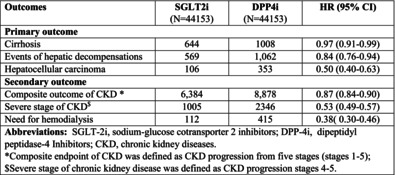

**Summary**

- In this population‐based cohort study, SGLT2 inhibitors (SGLT2i) were associated with significantly lower incidences of cirrhosis, hepatic decompensations (HR 0.84), and hepatocellular carcinoma (HCC) compared to DPP4i.
- Patients on SGLT2i demonstrated a notably reduced progression to chronic kidney disease (CKD) and severe CKD stages, as well as a lower need for hemodialysis compared to DPP4i users.
- Overall, SGLT2 inhibitors in patients with MASLD and T2DM demonstrated reduced risks of liver complications and a favorable impact on renal outcomes.

## 1 Introduction

The prevalence of the recently renamed metabolic dysfunction associated steatotic liver disease (MASLD) as well as type 2 diabetes mellitus (T2DM) has been steadily increasing, presenting a significant challenge for populations worldwide [1, 2]. The intersection of these two conditions often leads to a complex therapeutic landscape [3] particularly when considering the potential impact of antidiabetic medications on liver and renal outcomes. The recent name change of the former non‐alcoholic fatty liver disease (NAFLD) to MASLD [4] highlights the necessity of metabolic risk factors like T2DM for MASLD diagnosis [5]. While the primary aim of T2DM management is glycemic control, it is increasingly recognized that the choice of antidiabetic drugs may have far‐reaching implications beyond blood sugar regulation, especially in patients with coexisting MASLD [6].

Sodium‐glucose cotransporter‐2 inhibitors (SGLT2i) have emerged as a promising class of antidiabetic medications [7], noted for their unique mechanism of action that targets renal glucose reabsorption [8]. In addition to their efficacy in glycemic control, SGLT2i have shown cardiovascular benefits, which have further increased their popularity [9]. However, the specific impact of these drugs on hepatic and renal outcomes in patients with MASLD and T2DM remains an area of active investigation.

Given the intricate relationship between diabetes, MASLD, and the risk of chronic kidney disease (CKD) [10, 11], understanding the differential effects of antidiabetic medications on these comorbid conditions is of paramount importance. The potential of SGLT2i to influence liver‐related outcomes, such as the progression of MASLD to metabolic‐associated steatohepatitis (MASH), cirrhosis, and hepatocellular carcinoma (HCC) [3], is particularly intriguing.

In this context, our study aims to illuminate the comparative effectiveness of SGLT2i against other antidiabetic medications, specifically dipeptidyl peptidase‐4 inhibitors (DPP4i), glucagon‐like peptide‐1 receptor agonists (GLP‐1RA) and other second‐or‐third‐line antidiabetic drugs, in mitigating adverse liver and renal outcomes in patients with MASLD and T2DM.

## 2 Methods

### 2.1 Study Design and Data Source

This large, population‐based, retrospective cohort study was conducted using the TriNetX research network (Cambridge, MA, USA). TriNetX is a federated multicenter research network that provides real‐time access to an anonymized data set from participating healthcare organizations' electronic health records (EHR). Details of the data source, quality checks, and diagnosis codes used (according to predefined International Classification of Diseases, Clinical Modification [ICD‐9 and 10] codes) for patient selection are described in eMethods. Details of the TriNetX network are described in previous studies [12, 13]. We followed the strengthening the reporting of observational studies in epidemiology (STROBE) reporting guideline.

### 2.2 Study Participants

We identified all adult (aged ≥ 20 years) patients with MASLD and T2DM who newly started treatment with non‐insulin antidiabetic drugs (SGLT2i, DPP4i, GLP‐1Ras, sulfonylureas, meglitinides, thiazolidinediones, and acarbose) between January 1, 2013, and September 31, 2022. We limited the study cohort to patients who were required to have at least 1 year of follow‐up before cohort entry (i.e., receiving their first antidiabetic prescription). Furthermore, to reduce reverse causality and detection bias, we included only those with more than 1 year of follow‐up after the start of the study.

The identification of MASLD at the study's outset was based on the presence of specific ICD‐9 and 10 codes. Patients diagnosed with other liver diseases with any ICD codes were excluded from the MASLD. Patients were excluded if they met any of the following criteria: chronic liver disease other than MASLD, including alcohol, viral, drug‐induced, autoimmune, and genetic diseases; liver cirrhosis; or clinical diagnosis of hepatic decompensation (such as esophageal varices or ascites); history of excessive alcohol use, alcohol abuse, or alcohol use disorder; or history of alcohol‐related disorders; HIV infection; solid organ transplantation; patients with estimated glomerular filtration rate (eGFR) < 30 mL/min/1.73 m^2^ (within 6 months before the cohort entry) and history of undergoing dialysis treatment. Finally, patients with follow‐up of less than 1 year after the cohort and patients with any history of adverse liver and renal events, or dialysis interventions prior to inclusion in the cohort or prior to the index event were also excluded. We further excluded patients with a history of treatment with insulin before their initial prescription for a non‐insulin antidiabetic drug (since insulin therapy at baseline is considered for patients with advanced disease); female patients with a history of polycystic ovary syndrome or gestational diabetes; or those with type 1 DM. Finally, we excluded the patients who had a diagnosis of cancer, organ transplantation or dialysis, and eGFR < 30 mL/min/1.73 m2 before cohort entry within 6 months. Patients were followed from 1 year after cohort entry until an incident adverse liver and renal outcome, switch to a comparator drug, any cause of death, or end of the study (31, 2022), whichever occurred first.

### 2.3 Drug Exposure

Study cohort consisted of new users of SGLT2i (canagliflozin, dapagliflozin, or empagliflozin), DPP4i (sitagliptin, saxagliptin, linagliptin, and alogliptin), GLP‐1RAs (dulaglutide, exenatide, liraglutide, lixisenatide, or semaglutide), and other second or third‐line antidiabetic drugs (thiazolidinediones, sulfonylureas, meglitinides, α‐glucosidase inhibitors, insulin, or a combination of antidiabetic drugs; or add‐on or switched to an antidiabetic drug) were included. Cohort entry was defined as the date of the first‐ever prescription for one of the drugs of interest (SGLT2i, DPP4i, GLP‐1RAs or other second‐or‐third‐line antidiabetic drugs) during the study period, and their respective first exposure was defined as the index event. We used a lag of 6 months for all exposures to minimize protopathic bias and allow for a minimum and sufficient latency period after cohort entry [14, 15].

### 2.4 Matching Process

We used a propensity score matching (PSM) method to compare the new users of SGLT2i with new users of GLP‐1RAs and other second‐ third‐line antidiabetic drugs. The PSM was performed using 1:1 to reduce the confounding effects. The covariates were adjusted in the PSM model for a priori‐identified potential confounders, such as age, sex, race/ethnicity, nicotine dependence, body mass index (BMI), T2DM, hypertension, hyperlipidemia, hypercholesterolemia, chronic respiratory disease, chronic renal diseases, blood pressure, CKD, peripheral vascular diseases, diabetes‐related microvascular complications, glomerular diseases, osteoporosis, sleep apnea, abnormal laboratory findings (glycated hemoglobin, serum cholesterol, low‐density lipoprotein [LDL], high‐density lipoprotein [HDL], and triglycerides [TG]), and intake of cardiovascular medications (Table 1). Logistic regression was performed to obtain the propensity scores, and a greedy nearest‐neighbor matching algorithm was used to perform the matching with a caliper of 0.1 pooled standard deviations (SD). The balancing of potential confounding variables was evaluated using standardized mean differences (SMD) with a threshold set a priori at 0.10. We used SMD to measure the magnitude of difference between the groups rather than the p‐value because of their insensitivity to sample size. Logistic regression was performed using both Python (Python Software Foundation, Wilmington, Delaware, United States) and R 3.4.4 software (R Foundation for Statistical Computing, Vienna, Austria) to ensure the outputs matched and the order of the rows in the covariate matrix was randomized to eliminate this bias.

**TABLE 1:** Baseline characteristics of new SGLT2i users versus matched new DPP4i users in patients with MASLD and type 2 diabetes.

Variables	Before the propensity score match			After the propensity score match		
	SGLT2i (*N* = 60 465)	DPP4i (*N* = 49 134)	SMD	SGLT2i (*N* = 44 153)	DPP4i (*N* = 44 153)	SMD
Age in years, mean ± SD	58.8 ± 12.3	60.4 ± 12.8	0.1217	59.5 ± 12.2	59.5 ± 12.8	0.0039
Sex, *n* (%), Female	30 834 (50.9)	28 159 (57.3)	0.1270	24 376 (55.2)	24 443 (55.4)	0.0031
Ethnicity, *n* (%)						
Hispanic or Latino	8507 (14.1)	7257 (14.8)	0.0199	6546 (14.8)	6590 (14.9)	0.0028
Race, *n* (%)						
White	42 326 (70.0)	33 911 (69.0)	0.0214	30 897 (69.9)	30 792 (69.7)	0.0052
Black or African Americans	6841 (11.3)	5378 (10.9)	0.0117	4773 (10.8)	4885 (11.0)	0.0081
Others	6626 (10.9)	5686 (11.5)	0.0194	4868 (11.0)	4828 (10.9)	0.0029
Nicotine dependence, *n* (%)	10 030 (16.5)	6606 (13.4)	0.0881	6697 (17.2)	6839 (17.6)	0.0096
BMI (kg/m^2^), mean ± SD	34.7 ± 6.88	33.5 ± 6.86	0.1765	34.2 ± 6.78	33.5 ± 6.85	0.0963
Comorbidities, *n* (%)						
Hypertension	46 598 (77.0)	33 795 (68.7)	0.1873	31 938 (72.3)	32 093 (72.6)	0.0079
Hyperlipidemia	38 336 (63.4)	26 583 (54.1)	0.1897	25 525 (57.8)	25 527 (57.8)	< 0.0001
Chronic lower respiratory disease	19 254 (31.8)	13 347 (27.1)	0.1027	12 594 (28.5)	12 711 (28.7)	0.0059
Hypothyroidism	11 643 (19.2)	8544 (17.3)	0.0483	7998 (18.1)	8061 (18.2)	0.0037
Cerebrovascular disease	7725 (12.7)	5520 (11.2)	0.0474	5068 (11.4)	5147 (11.6)	0.0056
Ischemic heart disease	17 999 (29.7)	11 117 (22.6)	0.1630	10 729 (24.3)	10 776 (24.4)	0.0025
Glomerular disease	1752 (2.8)	1401 (2.8)	0.0028	1245 (2.8)	1284 (2.9)	0.0053
Peripheral vascular diseases	4881 (8.0)	3221 (6.5)	0.0583	2982 (6.7)	3047 (6.9)	0.0058
Diabetic retinopathy	3605 (5.9)	2489 (5.0)	0.0393	2298 (5.2)	2370 (5.3)	0.0073
Diabetic vasculopathy	5313 (8.7)	2583 (5.2)	0.1385	2523 (5.7)	2565 (5.8)	0.0041
Glomerular diseases	1752 (2.8)	1401 (2.8)	0.0028	1245 (2.8)	1284 (2.9)	0.0053
Heart failure	9917 (16.4)	5001 (10.1)	0.1841	4856 (10.9)	4945 (11.2)	0.0064
Osteoporosis	3845 (6.3)	3086 (6.2)	0.0032	2758 (6.2)	2803 (6.3)	0.0042
Obstructive sleep apnea	18 262 (30.2)	9692 (19.7)	0.2439	9698 (21.9)	9643 (21.8)	0.0030
Cardiovascular medications, *n* (%)						
Beta‐blockers	29 071 (48.0)	19 536 (39.7)	0.1670	18 286 (42.0)	18 380 (42.3)	0.0044
Antiarrhythmics	31 515 (52.0)	19 021 (38.6)	0.2704	18 621 (42.8)	18 589 (42.7)	0.0015
Antilipemic agents	42 698 (70.5)	29 661 (60.3)	0.2148	28 014 (64.4)	28 037 (64.5)	0.0011
ACE inhibitors	28 330 (46.7)	19 589 (39.8)	0.1401	18 308 (42.1)	18 478 (42.5)	0.0079
Diuretics	29 580 (48.8)	20 334 (41.3)	0.1506	18 054 (43.0)	18 247 (43.5)	0.0093
Vitamin D supplement	941 (1.5)	446 (0.9)	0.0587	435 (1.0)	441 (1.0)	0.0014
Vitamin E supplement	2561 (4.2)	1760 (3.5)	0.0335	2561 (4.2)	1760 (3.5)	0.0335
Calcium channel blockers	20 348 (33.5)	13 526 (27.5)	0.1323	12 131 (28.9)	12 341 (29.4)	0.0110
Antihypertensives, other	14 829 (24.4)	8756 (17.8)	0.1639	8178 (19.5)	8289 (19.7)	0.0067
Antihypertensive combinations	1370 (2.2)	233 (0.4)	0.1544	261 (0.6)	233 (0.5)	0.0087
Antidiabetic medications, *n* (%)						
Metformin	40 521 (66.9)	28 857 (58.7)	0.1703	27 188 (62.5)	27 239 (62.6)	0.0024
Insulin	29 504 (48.7)	17 556 (35.7)	0.2655	17 144 (39.4)	17 154 (39.4)	0.0005
Glipizide	12 168 (20.0)	9313 (18.9)	0.0289	8554 (19.6)	8575 (19.7)	0.0012
Semaglutide	3766 (6.2)	850 (1.7)	0.2313	942 (2.2)	850 (2.0)	0.0152
Dulaglutide	7350 (12.1)	1705 (3.4)	0.3275	1772 (4.2)	1705 (4.0)	0.0080
Tirzepatide	113 (0.1)	38 (0.08)	0.0301	42 (0.1)	38 (0.1)	0.0031
Liraglutide	6625 (10.9)	1966 (4.0)	0.2663	2071 (4.9)	1965 (4.6)	0.0118
Pioglitazone	4872 (8.0)	3674 (7.4)	0.0213	3363 (7.7)	3376 (7.7)	0.0011
Glyburide	3231 (5.3)	3016 (6.1)	0.0345	2554 (5.8)	2546 (5.8)	0.0008
Repaglinide	676 (1.1)	528 (1.1)	0.0040	461 (1.1)	475 (1.1)	0.0031
Rosiglitazone	384 (0.6)	477 (0.9)	0.0377	339 (0.7)	344 (0.7)	0.0013
Acarbose	296 (0.4)	235 (0.4)	0.0015	217 (0.4)	212 (0.4)	0.0016
Nateglinide	262 (0.4)	221 (0.4)	0.0026	207 (0.4)	198 (0.4)	0.0030
Ertugliflozin	194 (0.3)	87 (0.1)	0.0288	108 (0.2)	87 (0.2)	0.010
Alogliptin	486 (0.8)	331 (0.6)	0.0151	328 (0.7)	317 (0.7)	0.0029

Abbreviations: ACE, angiotensin‐converting‐enzyme; BMI, body mass index; MASLD, nonalcoholic fatty liver disease; SD, standard deviation; SGLT2, sodium‐glucose cotransporter‐2; SMD, standard mean difference.

### 2.5 Outcome

The primary outcome was to assess the incidence of a new onset of adverse liver events as a first incidence, which were categorized as (1) cirrhosis, (2) composite outcome of hepatic decompensation events, and (3) HCC. A composite outcome of hepatic decompensation events was defined as the first occurrence of any one of the following events: variceal hemorrhage, ascites, hepatic encephalopathy [16]. The secondary outcome was to evaluate the incidence of adverse renal outcomes, which were categorized as (1) CKD, (2) composite endpoint of a severe stage of CKD, and (3) Need for renal hemodialysis. A composite outcome of CKD was defined as CKD progression from five stages (stages 1–5), whereas a composite endpoint of a severe stage of CKD was defined as CKD progression stages 4–5.

### 2.6 Statistical Analyses

All analyses were performed using the TriNetX real‐time analytics platform. This approach involves dynamic and immediate data analysis, enabling continuous processing and interpretation of data as it is generated. Categorical variables were compared using the Pearson chi‐square test, and continuous variables were compared using an independent‐sample t‐test. Continuous variables were expressed as mean ± SD, and categorical variables were presented as frequency and percentage. Analyses were performed to examine the rate of adverse CVEs using Cox proportional hazards models. Hazard ratios (HRs) and confidence intervals (CIs), along with tests for proportionality, were calculated using R's Survival package v3.2‐3. The results were validated by comparing them with the output from SAS version 9.4. Patients were censored when the time window ended or the day after the last fact in their record. We utilized a 1:1 propensity matching strategy to establish comparable groups of patients treated with different antidiabetic drugs, including SGLT2i, DPP4i, and GLP1RA. In addition, we used this matching approach aimed to balance the covariates between the groups effectively. To account for clustering within the 1:1 propensity‐matched sample and address the loss of independence among individuals resulting from the matching procedure, we incorporated a robust variance estimator in the Cox regression model [17]. The robust variance estimator is essential in enhancing the accuracy of our analytical approach and ensuring the validity of the study's findings. A priori‐defined two‐sided alpha of < 0.05 was used for statistical significance.

### 2.7 Ancillary Analysis

GLP‐1RAs may directly improve hepatic steatosis due to their action on upregulating fatty acid metabolism and insulin signaling pathways, and GLP‐1RAs provide benefit to patients with MASLD and provide histological improvements in steatohepatitis for patients with NASH [18]. Hence, we used GLP1RA as a control in the secondary analysis. In this analysis, we matched new users of SGLT2i to new GLP1RA users on propensity scores.

### 2.8 Sensitivity Analyses

We conducted two sensitivity analyses to ensure the robustness of our findings due to the heterogeneous nature of study outcomes. The first sensitivity analysis evaluated the influence of different reference groups/disease stages. We compared the use of SGLT2i (second‐or‐third‐line antidiabetic drugs) with second or third‐line antidiabetic therapy to minimize confounding, thereby allowing us to explore the impact of including a reference group with the same disease stage, even though we adjusted for microvascular complications. In the second sensitivity analysis, we estimated the rates of new incidences of study outcomes by excluding patients with outcomes 2 years after the index event. Both analyses were performed using the same methods identical to the primary analysis. Our reporting of the results follows the EQUATOR guidelines.

## 3 Results

### 3.1 Baseline Characteristics

**FIGURE 1:**
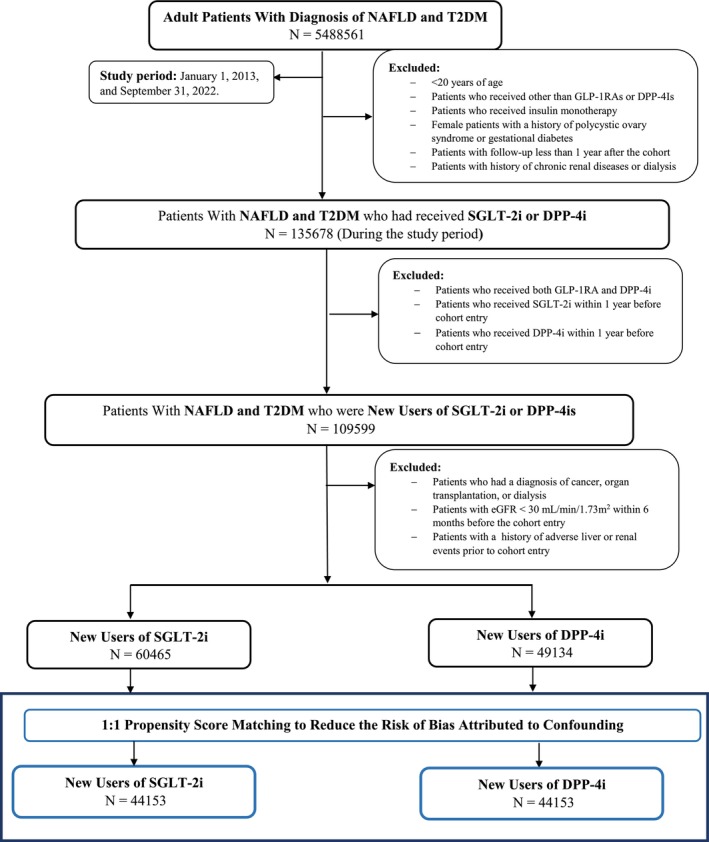
Study flowchart: study flow chart of patient selection in the study cohort for new users of sodium‐glucose cotransporter‐2 inhibitors versus glucagon‐like peptide‐1 receptor agonist and new users of dipeptidyl peptidase 4 (DPP‐4i) inhibitors (active‐comparator). DPP‐4i, dipeptidyl peptidase 4 inhibitors; eGFR, estimated glomerular filtration rate; MASLD, nonalcoholic fatty liver disease; SGLT‐2i, sodium‐glucose cotransporter‐2 inhibitors; T2DM, type 2 diabetes mellitus.

Table 1 provides a comparison of the baseline characteristics between new users of SGLT2i and those starting on DPP4i in a cohort of patients with MASLD and T2DM. The study flow diagram is shown as Figure 1. Before propensity matching (Figure S1), there were some notable differences between groups. The SGLT2i users were slightly younger on average compared to the DPP4i users (58.8 vs. 60.4 years, Table 1) and had a lower proportion of female patients compared to the DPP4i group (50.9% vs. 57.3%, Table 1). In terms of ethnicity and race, both groups were predominantly White, with a slightly higher percentage of Hispanic or Latino patients in the DPP4i group. Another notable difference was in nicotine dependence, which was more prevalent in the SGLT2i group (16.5%) than in the DPP4i group (13.4%, Table 1). In terms of BMI, the SGLT2i users had a marginally higher average BMI than the DPP4i users (34.7 vs. 33.5). The prevalence of comorbidities such as hypertension, hyperlipidemia, and heart failure was also higher in the SGLT2i group before matching. This was mirrored in the use of related medications; for instance, the SGLT2i users were more likely to be on beta‐blockers, antilipemic agents, and diuretics (Table 1). However, after applying PSM, these initial differences were resolved (Figure S1).

### 3.2 SGLT2i Reduces Hepatic and Kidney Outcomes Compared to DDP4i

In the SGLT2i group, cirrhosis was observed in 644 patients, compared to 1008 in the DPP4i group, yielding a HR of 0.97 (95% CI: 0.91–0.99, *p* < 0.001). Regarding hepatic decompensations, the SGLT2i group experienced 569 events, markedly fewer than the 1062 events in the DPP4i group, with an HR of 0.84 (95% CI: 0.76–0.94, *p* < 0.001, Table 2). Most notably, the incidence of HCC was substantially lower in the SGLT2i group (106 events) compared to the DPP4i group (353 events), with an HR of 0.50 (95% CI: 0.40–0.63, *p* < 0.001).

**TABLE 2:** Main Outcomes: Cardiovascular outcomes between the new users of SGLT2i versus DPP4i inhibitors in patients with MASLD and T2DM.

Outcomes	SGLT2i (*N* = 44 153)	DPP4i (*N* = 44 153)	HR (95% CI)
Primary outcome			
Cirrhosis	644	1008	0.97 (0.91–0.99)
Events of hepatic decompensations	569	1062	0.84 (0.76–0.94)
Hepatocellular carcinoma	106	353	0.50 (0.40–0.63)
Secondary outcome			
Composite outcome of CKD*	6384	8878	0.87 (0.84–0.90)
Severe stage of CKD^$^	1005	2346	0.53 (0.49–0.57)
Need for hemodialysis	112	415	0.38 (0.30–0.46)

Abbreviations: CKD, chronic kidney diseases; DPP‐4i, dipeptidyl peptidase‐4 Inhibitors; SGLT‐2i, sodium‐glucose cotransporter 2 inhibitors.

* Composite endpoint of CKD was defined as CKD progression from five stages (stages 1–5).

^$^ Severe stage of chronic kidney disease was defined as CKD progression stages 4–5.

CKD was notably lower in the SGLT2i group (6384 events) than in the DPP4i group (8878 events), resulting in an HR of 0.87 (95% CI: 0.84–0.90, *p* < 0.001, Table 2). When focusing on severe stages of CKD (stages 4–5), the SGLT2i group had 1005 events compared to 2346 in the DPP4i group, with an HR of 0.53 (95% CI: 0.49–0.57, *p* < 0.001), suggesting a significantly lower progression to severe CKD stages. Furthermore, the need for hemodialysis was markedly lower in the SGLT2i group (112 events) compared to the DPP4i group (415 events), with an HR of 0.38 (95% CI: 0.30–0.46, *p* < 0.001).

### 3.3 SGLT2i Reduces Hepatic Outcomes Compared to GLP‐1RA

In our ancillary analysis, we compared liver and renal outcomes between new users of SGLT2i and GLP‐1RA, each group comprising 56 398 patients. We observed cirrhosis in 830 patients in the SGLT2i group and 944 in the GLP‐1RA group, leading to an HR of 0.94 (95% CI: 0.86–1.04). Regarding hepatic decompensations, the incidence was comparable between the SGLT2i (740 events) and GLP‐1RA (794 events) groups, with an HR of 1.01 (95% CI: 0.91–1.12). Similarly, the risk of HCC appeared almost identical between the groups, with 133 events in the SGLT2i group and 131 in the GLP‐1RA group, yielding an HR of 1.10 (95% CI: 0.86–1.40, Table 3).

**TABLE 3:** Ancillary analysis: Liver and Renal outcomes between the new users of SGLT2i versus GLP‐1RA among patients with MASLD and T2DM.

Outcomes	SGLT2i (*N* = 56 398)	GLP‐1RA (*N* = 56 398)	HR (95% CI)
Primary outcome			
Cirrhosis	830	944	0.94 (0.86–1.04)
Events of hepatic decompensations	740	794	1.01 (0.91–1.12)
Hepatocellular carcinoma	133	131	1.10 (0.86–1.40)
Secondary outcome			
Composite outcome of CKD*	2568	2884	0.92 (0.87–0.97)
Severe stage of CKD^$^	695	960	0.77 (0.70–0.85)
Need for hemodialysis	166	253	0.71 (0.58–0.87)

Abbreviations: CKD, chronic kidney disease; GLP‐1RA, glucagon‐like peptide‐1 receptor agonists; SGLT‐2i, sodium‐glucose cotransporter 2 inhibitors.

* Composite endpoint of CKD was defined as CKD progression from five stages (stages 1–5).

^$^ Severe stage of chronic kidney disease was defined as CKD progression stages 4–5.

For CKD, the SGLT2i group showed 2568 events compared to 2884 in the GLP‐1RA group, resulting in an HR of 0.92 (95% CI: 0.87–0.97). The difference was more pronounced in the severe stages of CKD, with the SGLT2i group experiencing 695 events versus 960 in the GLP‐1RA group, corresponding to an HR of 0.77 (95% CI: 0.70–0.85). Furthermore, the need for hemodialysis was significantly lower in the SGLT2i group (166 events) compared to the GLP‐1RA group (253 events), with an HR of 0.71 (95% CI: 0.58–0.87).

### 3.4 SGLT2i Reduces Hepatic and Kidney Outcomes Compared to Second‐Or‐Third‐Line Antidiabetic Medications

In the Sensitivity Analysis, we explored liver and renal outcomes between new users of SGLT‐2i versus those on other second‐or‐third‐line anti‐diabetic medications, each group with 55 173 patients (Table 4). In the group treated with SGLT‐2i, there were 921 cases of cirrhosis compared to 1478 in the group on other second‐or‐third‐line medications, resulting in a HR of 0.83 (95% CI: 0.75–0.91). This suggests a significantly reduced risk of cirrhosis associated with SGLT‐2i. For hepatic decompensations, 802 events were recorded in the SGLT‐2i group, lower than the 1289 events in the second‐or‐third‐line group, yielding an HR of 0.86 (95% CI: 0.78–0.94). Additionally, HCC was less prevalent in the SGLT‐2i group with 129 events, compared to 289 events in the second‐or‐third‐line group, with an HR of 0.93 (95% CI: 0.75–0.98) (Table 4).

**TABLE 4:** Sensitivity Analysis 1: Liver and renal outcomes between the new users of SGLT2I versus other second‐or‐third‐line anti‐diabetic medications patients with MASLD and T2DM.

Outcomes	SGLT‐2i (*N* = 55 173), *n*	Second‐or‐third line (*N* = 55 173), *n*	HR (95% CI)
Primary outcome			
Cirrhosis	921	1478	0.83 (0.75–0.91)
Events of hepatic decompensations	802	1289	0.86 (0.78–0.94)
Hepatocellular carcinoma	129	289	0.93 (0.75–0.98)
Secondary outcome			
Composite outcome of CKD*	2514	4816	0.85 (0.81–0.90)
Severe stage of CKD^$^	678	1696	0.70 (0.64–0.77)
Need for hemodialysis	145	494	0.55 (0.46–0.67)

Abbreviations: CKD, chronic kidney disease; SGLT‐2i, sodium‐glucose cotransporter 2 inhibitors.

* Composite endpoint of CKD was defined as CKD progression from five stages (stages 1–5).

^$^ Severe stage of chronic kidney disease was defined as CKD progression stages 4–5.

For CKD, the SGLT‐2i group experienced 2514 events, markedly fewer than the 4816 events in the second‐or‐third line group, with an HR of 0.85 (95% CI: 0.81–0.90). In the context of severe CKD stages, the SGLT‐2i group had 678 events, significantly lower than the 1696 events in the second‐or‐third line group, resulting in an HR of 0.70 (95% CI: 0.64–0.77). In addition, hemodialysis was significantly less common in the SGLT‐2i group, with 145 events compared to 494 in the other drug group, giving an HR of 0.55 (95% CI: 0.46–0.67).

### 3.5 SGLT2i Reduce Hepatic and Kidney Outcomes Compared to DDP4i More Than 2 Years After Initiation

In another Sensitivity Analysis, we compared new users of SGLT‐2 with those using DPP‐4i and excluded outcomes from the first 2 years of treatment for each patient group. In the SGLT‐2i group, cirrhosis was observed in 286 patients, considerably lower than the 595 cases in the DPP‐4i group, resulting in a HR of 0.95 (95% CI: 0.93–0.98). Hepatic decompensations occurred in 254 patients in the SGLT‐2i group, compared to 623 in the DPP‐4i group, yielding an HR of 0.90 (95% CI: 0.78–0.99). Furthermore, the incidence of HCC was lower in the SGLT‐2i group, with 47 events versus 114 in the DPP‐4i group, suggesting a reduced risk with an HR of 0.92 (95% CI: 0.65–0.97). For the composite outcome of CKD, there were 1338 events in the SGLT‐2i group and 2107 in the DPP‐4i group, indicating a reduced CKD progression with an HR of 0.88 (95% CI: 0.82–0.96). In terms of advancing to severe stages of CKD, the SGLT‐2i group showed 506 events, notably fewer than the 806 events in the DPP‐4i group, resulting in an HR of 0.61 (95% CI: 0.53–0.71). Additionally, the need for hemodialysis was significantly lower in the SGLT‐2i group (120 events) compared to the DPP‐4i group (238 events), with an HR of 0.57 (95% CI: 0.43–0.76).

## 4 Discussion

The findings of our study suggest that the use of SGLT2i in patients with MASLD and T2DM may lead to a reduction in liver disease progression. This observation aligns with emerging evidence indicating the potential hepatic benefits of SGLT2i, which extend beyond their primary role in glycemic control [19]. First, the role of SGLT2i in modulating insulin sensitivity and reducing hyperglycemia is well‐established [20]. Glycemic control is crucial in patients with MASLD, where insulin resistance plays a pivotal role in disease pathogenesis [8]. Even though insulin sensitivity might not be affected by SGLT2i [21], the potential reduction of visceral adipose tissue and also hepatic gluconeogenesis [22], SGLT2i may directly and indirectly mitigate vital effects of MASLD progression. SGLT2 is expressed in the hepatocytes of patients with chronic liver disease and is associated with various factors including inflammation and disease severity [23]. Another potential mechanism is the impact of SGLT2i on the renin‐angiotensin‐aldosterone system (RAAS) [24]. Abnormal RAAS activation is implicated in the pathogenesis of MASLD [25]. SGLT2i may attenuate this activation, thereby exerting an anti‐inflammatory and antifibrotic effect on the liver [8]. Lastly, SGLT2i have demonstrated well‐established cardiovascular benefits, including reductions in systemic inflammation [25, 26]. These systemic effects could indirectly benefit liver disease progression.

The protective effects of SGLT2i on the liver have only recently been established, and the protective effects on the kidneys are well known: Both GLP1‐RA and SGLT2i are known to reduce the risk of kidney‐related outcomes in patients with diabetes [27]. Still, it is an interesting observation that SGLT2i seem superior to GLP1‐RA in preventing liver disease progression in our study. Studies encompassing a diverse range of methodologies, including an MVP target emulation trial [27], a Scandinavian cohort study, and an Italian randomized trial [28] all compared SGLT2i and GLP‐1RA independent of their MASLD status and also found that SGLT2i have a stronger kidney‐related effect than GLP‐1RA [29].

The renal protective mechanisms of GLP‐1RAs predominantly manifest through a reduction in macroalbuminuria [30]. However, a recent meta‐analysis indicated that excluding macroalbuminuria from the evaluation yielded a minor 8% relative reduction in renal outcomes for diabetic patients on GLP‐1RAs [31]. Conversely, SGLT2i demonstrated a substantial 45% relative risk reduction in a composite outcome of declining eGFR, end‐stage renal disease, and renal‐related death, in line with our findings in the MASLD cohort [32].

While the precise pathophysiological pathways through which these drug classes confer renal protection remain a subject of ongoing research, it is apparent that both classes achieve modest and comparable reductions in HbA1c [33, 34, 35]. Thus, their cardiovascular and renal benefits seem to extend beyond glucose control, attributed to their distinct pleiotropic properties. Notably, the natriuresis and inhibition of tubular and glomerular feedback facilitated by SGLT2i are hypothesized to play a central role [36]. These mechanisms could account for the observed reduction in hospitalization for heart failure and the deceleration of diabetic kidney disease progression, aspects particularly pertinent to patients with MASLD [37].

Our study's insights must be contextualized within its inherent limitations. Primarily, the observational design precludes causal inferences. Additionally, the reliance on EHRs introduces the potential for coding inaccuracies and data entry errors, despite efforts to mitigate these through standardized case identification. Residual confounding remains a possibility, despite rigorous adjustments. The use of the TriNetX database further limits our study in several ways. First, TriNetX does not provide access to raw patient‐level data, which restricts our ability to perform sensitivity analyses for missing data or competing risk analyses. Second, the platform aggregates data from multiple healthcare systems, which may introduce variability in diagnostic coding, medication records, and laboratory measurements. However, standardized measures were implemented to identify cases and minimize documentation errors, including using an AASLD‐proposed validated diagnostic algorithm to identify individuals with MASLD [38]. Finally, the absence of histological confirmation in our inclusion criteria may have led to some misclassification, and we acknowledge that future studies are needed to link SGLT2i to the reduction of steatohepatitis. Furthermore, the specific patient population with MASLD and T2DM might have unique characteristics that influence the efficacy and safety profile of SGLT2i.

In conclusion, our study adds to the growing body of literature suggesting that SGLT2i may offer benefits in reducing liver disease progression in patients with MASLD and T2DM. The potential mechanisms underlying these benefits may include improved glycemic control, reduction in body weight and visceral adiposity, modulation of the RAAS, systemic cardiovascular effects, and favorable changes in lipid metabolism. Still, future research, particularly randomized controlled trials, is needed to further elucidate these relationships and optimize therapeutic strategies for this growing patient population.

## Author Contributions

A.K. and S.A.A. contributed to the concept of the study and study design. A.K. was responsible for data acquisition and statistical analysis. A.K., C.V.S., and D.M. drafted the manuscript. All authors were involved with the interpretation of the data and critically revised the manuscript for important intellectual content. A.K. provided technical support. S.A.A. provided administrative, material support, and supervised the project. All authors reviewed and approved the final version of the manuscript. A.K. is the guarantor of this work and, as such, had full access to all the data in the study and takes responsibility for the integrity of the data and the accuracy of the data analysis.

## Conflicts of Interest

The authors declare no conflicts of interest.

## Supporting information


**Data S1** Supporting Information.

## Data Availability

The authors have nothing to report.
