# Supplementary material for: Adverse Liver and Renal Outcomes After Initiating SGLT‐2i and GLP‐1RA Therapy Among Patients With Diabetes and MASLD
Source: J Diabetes. 2025 Apr 27;17(4):e70069. doi: 10.1111/1753-0407.70069 (PMC12034490; doi:10.1111/1753-0407.70069)
Supplement: Supplementary file 1 — Data S1 Supporting Information. [file JDB-17-e70069-s001.doc]

**Electronic Supplementary Material (ESM) Online Content**

**Appendix.** Supplementary Methods

**Data Source**

We used TriNetX (Cambridge, MA, USA), a global federated health research network providing real-time access to electronic health records (EHRs). TriNetX platform de-identifies and aggregates EHR data from 66 healthcare organizations (HCOs), most of which are large academic medical institutions with both inpatient and outpatient facilities at multiple locations across 50 states in the United States. The Real-time access to health insurance portability and accountability act– de-identified, compliant, and longitudinal clinical data to member HCOs is provided cloud-based. The de-identified clinical data, such as diagnoses, procedures, medications, laboratory values, and genomic information, are continuously aggregated directly from the EHR of the participating HCOs. Participating HCOs include outpatient, inpatient, and specialty care services and provide care to a diverse patient population from different ethnicity, age groups, geographical region, and income levels. Both the patients and HCOs, as data sources, stay anonymous. The use of TriNetX has several inherent limitations. First, the platform aggregates data from multiple healthcare systems, which may introduce variability in diagnostic coding, medication records, and laboratory measurements. Second, TriNetX does not provide access to raw patient-level data, limiting our ability to perform certain analyses, such as sensitivity analyses for missing data or competing risk analyses. Despite these limitations, we implemented rigorous data cleaning and harmonization procedures to ensure the reliability of our dataset."

As a federated network, TriNetX data have been granted a waiver from the Western institutional review board since only aggregated counts and statistical summaries of de-identified information without any protected health information were received from participating HCOs. In addition, no study-specific activities are performed in retrospective analyses.

**Standardizing the terminology and data quality check:**

The TriNetX software verifies the basic formatting to confirm that data are appropriately characterized. Patient counts were rounded up to the nearest 10 in our analysis to safeguard protected health information. TriNetX has production capabilities that have been tested that map data extensively from each of these structures to the standard model within TriNetX and can extract details of interest from the narrative content of clinical documents using natural language processing. The contributing EHR systems used United Medical Language System (UMLS) for coding. TriNetX maps the data to a standard and controlled set of clinical terminologies, for example, mapping disease terms from Systematized Nomenclature of Medicine—Clinical Terms (SNOMED CT) to International Classification of Diseases, and Clinical Modification (ICD-9 and 10 CM), drug terms from National Drug Codes (NDCs) to RxNorm. TriNetX enforces a list of required fields (e.g., patient identifier) and rejects those records where the required data is lacking. Referential integrity checking confirms that data spanning multiple database tables can be successfully joined together. TriNetX requires at least 1 non-demographic fact for a patient to be calculated in a given data set. Patient records with only demographic information are not included in data sets. As the data are refreshed, the TriNetX software monitors change in data volumes over time to ensure data validity.

**Selection of Patients:**

The search was conducted following the criteria provided by TriNetX to identify potential patients. These codes included the ICD-9, 10 CM. We combined patients into a single cohort of NAFLD/NASH defined by a diagnosis of NAFLD based on ( 571.8, K76.0, and K75.81). 571.5 , K74.6 Other and unspecified cirrhosis othe f liver.

**Exclusion criteria:**

We excluded the patients if they had a diagnosis of another defined cause of liver disease other than NAFLD, including acute alcohol abuse or chronic alcohol abuse, alcoholic liver disease, toxic liver disease, viral hepatitis, Wilson's disease, autoimmune hepatitis, Gaucher disease, primary biliary cholangitis, hemochromatosis, primary sclerosing cholangitis. Diagnosis codes based on the exclusion criteria are listed below. 070, Viral hepatitis; 571.6, 576.1, Autoimmune liver disease (AIH, PBC, PSC); 275.0, Hemochromatosis; 275.1, Wilson’s disease; 277.6, Alpha-1-antitrypsin deficiency; 453.0, Budd-Chiari syndrome; 571.4, Chronic hepatitis, unspecified; 571.6, Secondary or unspecified biliary cirrhosis, 303, 305.0, alcohol use disorder; 291, 357.5, 425.5, 535.3, 980.1, 980.9 somatic consequences of alcohol (except ALD); 305.1-9, drug use disorders except nicotine/caffeine; 571.5, Cirrhosis, compensated; 456.1, 456.21, Esophageal varices, not bleeding; 456.0, 456.20, Esophageal varices, bleeding; 789.5, Ascites; 572.2, hepatic encephalopathy; 572.4, Hepatorenal syndrome; 572.3, Portal hypertension; V427, Liver transplantation status, 572.8, Chronic or unspecified liver failure; 570, Acute or subacute liver failure; 571,9, Hepatic fibrosis or sclerosis or fibrosis with sclerosis. K72 Acute and subacute hepatic failure without coma necrosis; K76.2 Central hemorrhagic necrosis of liver necrosis; K700 Alcoholic fatty liver hepatitis; K7010 Alcoholic hepatitis without ascites hepatitis; K7030 Alcoholic cirrhosis. Of liver without ascites cirrhosis. K709 Alcoholic liver disease, unspecified cirrhosis; K730 Chronic persistent hepatitis, not elsewhere classified hepatitis; K732 Chronic active hepatitis, not elsewhere classified hepatitis; K738 Other chronic hepatitis, not elsewhere classified hepatitis; K739 Chronic hepatitis, unspecified hepatitis; K740 Hepatic fibrosis; K74.1 Hepatic sclerosis cirrhosis; K743 Primary biliary cirrhosis;K74.4 Secondary biliary cirrhosis;K74.5 Biliary cirrhosis, unspecified cirrhosis;;K75.4 Autoimmune hepatitis;K76.7 Hepatorenal syndrome cirrhosis;K77 Liver disorders in diseases classified elsewhere hepatitis;K71.6 Toxic liver disease with hepatitis, not elsewhere classified hepatitis;B17.0 Acute delta-(super) infection of hepatitis B carrier hepatitis;B1710 Acute hepatitis C without hepatic coma hepatitis;B17.2 Acute hepatitis E hepatitis;B17.8 Other specified acute viral hepatitis;B18.2 Chronic viral hepatitis C hepatitis;B18.8 Other chronic viral hepatitis;B18.9 Chronic viral hepatitis, unspecified hepatitis;B0081 Herpesviral hepatitis hepatitis;B15.0 Hepatitis A with hepatic coma hepatitis;B15.9 Hepatitis A without hepatic coma hepatitis;B16.0 Acute hepatitis B with delta-agent with hepatic coma hepatitis;B16.1 Acute hepatitis B with delta-agent without hepatic coma hepatitis;B16.2 Acute hepatitis B without delta-agent with hepatic coma hepatitis;B16.9 Acute hepatitis B without delta-agent and without hepatic coma hepatitis;B17.11 Acute hepatitis C with hepatic coma hepatitis;B17.2 Acute hepatitis E hepatitis;B17.8 Other specified acute viral hepatitis;B179 Acute viral hepatitis, unspecified hepatitis;B18.0 Chronic viral hepatitis B with delta-agent hepatitis;B18.1 Chronic viral hepatitis B without delta-agent hepatitis;B18.2 Chronic viral hepatitis C hepatitis;B19.0 Unspecified viral hepatitis with hepatic coma hepatitis;B19.10 Unspecified viral hepatitis B without hepatic coma hepatitis;B19.11 Unspecified viral hepatitis B with hepatic coma hepatitis; B19.20 Unspecified viral hepatitis C without hepatic coma hepatitis; B19.21 Unspecified viral hepatitis C with hepatic coma hepatitis; B19.9 Unspecified viral hepatitis without hepatic coma hepatitis; B25.1 Cytomegaloviral hepatitis; B25.1 Cytomegaloviral hepatitis; B26.81 Mumps hepatitis; B58.1 Toxoplasma hepatitis; B942 Sequelae of viral hepatitis; K70.11 Alcoholic hepatitis with ascites hepatitis; E83.01, Wilson's disease; K76.9 Liver disease, unspecified hepatitis; K72.90 Hepatic failure, unspecified without coma cirrhosis; K73.1 Chronic lobular hepatitis, not elsewhere classified hepatitis; K75.2 Nonspecific reactive hepatitis; B20, human immunodeficiency virus; K75.3 Granulomatous hepatitis, not elsewhere classified hepatitis; K75.89 Other specified inflammatory liver diseases hepatitis; K70.31 Alcoholic cirrhosis of liver with ascites cirrhosis; F10.2 Alcohol dependence; K71.7 Toxic liver disease with fibrosis and cirrhosis of the liver.

**Baseline characteristics related codes:**

Hypertension, E11.1, Type 2 diabetes; I60-I69 Cerebrovascular diseases; K21, Gastroesophageal reflux disease; J40-J47, Chronic respiratory diseases; G47.33 Obstructive sleep apnea; N18 Chronic kidney diseases; M81 Osteoporosis; E11.21 Nephropathy; E11.31 Retinopathy; E11.42 Polyneuropathy; N00-N08 Glomerular diseases.

**Diagnosis and procedure codes to assist in identifying cardiovascular conditions:**

155.0, hepatocellular carcinoma; 155.2, Liver cancer, unspecified; C22.0 Liver cell carcinoma; R18 Ascites; varices I85.0, I85.9, I86.4, I98.2, I98.3; I85.0 Oesophageal varices with bleeding; I85.9 Oesophageal varices without bleeding; I86.4 Gastric varices; I98.2 Oesophageal varices without bleeding in diseases classified elsewhere; I98.3 Oesophageal varices with bleeding in diseases classified elsewhere; spontaneous bacterial peritonitis K65.0, K65.9; K65.0 Acute peritonitis; K65.9 Peritonitis, unspecified; G93.4 Encephalopathy, unspecified; hepatic encephalopathyG31.2, G93.4; K74.6 Other and unspecified cirrhosis of liver; Z49 Encounter for care involving renal dialysis; N18 Chronic kidney disease; N18.1 Chronic kidney disease, stage 1; N18.2 Chronic kidney disease, stage 2 (mild); N18.3 Chronic kidney disease, stage 3 (moderate); N18.30 Chronic kidney disease, stage 3 unspecified; N18.31 Chronic kidney disease, stage 3a; N18.32 Chronic kidney disease, stage 3b; N18.4 Chronic kidney disease, stage 4 (severe); N18.5 Chronic kidney disease, stage 5; N18.6 End stage renal disease; N18.9 Chronic kidney disease, unspecified.


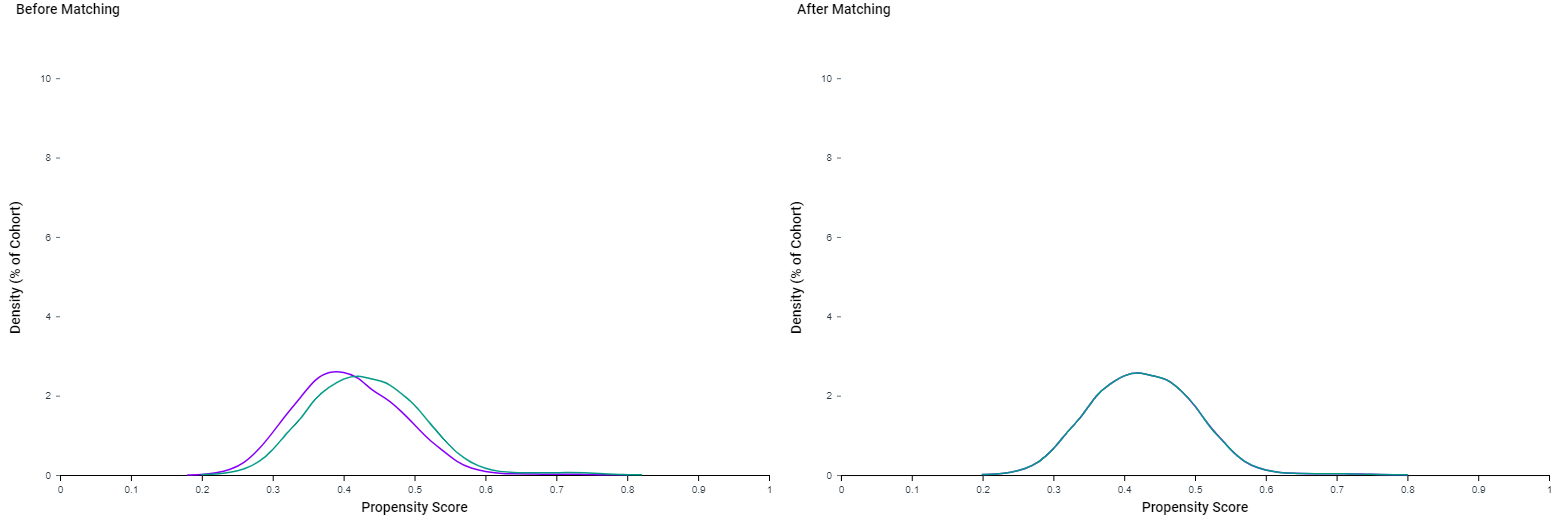


**Supplementary Figure 1:** Propensity score density graph for the users of sodium-glucose cotransporter-2 inhibitors (Purple line) versus dipeptidyl peptidase4 inhibitors (Green line) users among patients with metabolic dysfunction-associated steatotic liver disease and type 2 diabetes before and after propensity score matching.

**Supplemental** Table 1: Sensitivity Analysis 2: Liver and renal outcomes between the new users of SGLT2I vs. DPP-4i after excluding first 2 years outcomes among patients with MASLD and T2DM

| **Outcomes** | **SGLT-2i**  (N=44153), n | **DPP-4i**  (N=44153), n | **HR (95% CI)** |
| --- | --- | --- | --- |
| **Primary outcome** | | | |
| Cirrhosis | 286 | 595 | 0.95 (0.93-0.98) |
| Events of hepatic decompensations | 254 | 623 | 0.90 (0.78 - 0.99) |
| Hepatocellular carcinoma | 47 | 114 | 0.92 (0.65- 0.97) |
| **Secondary outcome** | | | |
| Composite outcome of CKD * | 1338 | 2107 | 0.88 (0.82 - 0.96) |
| Severe stage of CKD^$^ | 506 | 806 | 0.61 (0.53 - 0.71) |
| Need for hemodialysis | 120 | 238 | 0.57 (0.43 - 0.76) |
| **Abbreviations:** SGLT-2i, sodium-glucose cotransporter 2 inhibitors; DPP-4i, dipeptidyl peptidase-4 Inhibitors; CKD, chronic kidney diseases.  *Composite endpoint of CKD was defined as CKD progression from five stages (stages 1-5); $Severe stage of chronic kidney disease was defined as CKD progression stages 4-5. | | | |
